# Reducing umbilical catheter migration rates by using a novel securement device

**DOI:** 10.1038/s41372-024-01943-1

**Published:** 2024-03-23

**Authors:** Juliana R. Perl, Tanya Crabtree-Beach, Amy Olyaei, Madeline Hedges, Brian K. Jordan, Brian Scottoline

**Affiliations:** 1 Stanford Byers Center for Biodesign, Stanford University, Stanford, CA 94305 USA; 2 Division of Neonatology, Department of Pediatrics, Oregon Health & Science University, Portland, OR 97239 USA

**Keywords:** Paediatrics, Bacterial infection, Outcomes research, Disease prevention

## Abstract

**Objective:**

This study evaluates the effectiveness of a novel device, LifeBubble, in reducing umbilical cord catheter (UC) migration and associated complications in neonates.

**Study design:**

A retrospective review was performed at Oregon Health & Science University’s NICU (2019–2021) to compare standard adhesive securement with LifeBubble. The primary outcomes were UC migration, discontinuation due to malposition, and CLABSI incidence. Differences between groups were statistically analyzed and logistic regression used to adjust for potential confounders.

**Results:**

Among 118 neonates (57 LifeBubble, 61 adhesive), LifeBubble significantly reduced migration of any UC > 1 vertebral body (12.3% vs. 55.7%), including UVC migration (5.3% vs. 39.3%) and UAC migration (7.0% vs 23.0%), as well as UVC discontinuation due to malposition (5.6% vs 37.7%). The number needed to treat (NNT) to prevent one instance of UVC discontinuation is 4.

**Conclusion:**

LifeBubble effectively reduces UC migration and premature discontinuation, indicating its potential to enhance neonatal care and safety.

## Introduction

Umbilical cord catheters (UCs) are vital tools for monitoring and providing essential support to critically ill newborns in neonatal intensive care units (NICUs). These catheters include both umbilical vein catheters (UVCs) and umbilical artery catheters (UACs), which allow for the delivery of parenteral nutrition, fluids, and therapeutics (UVCs and UACs) as well as the continuous monitoring of arterial blood pressure and arterial blood gas sampling (UACs) [1]. UCs are essential to neonatal care, as they can be placed in relatively large and easily accessible vessels, while other percutaneous central access techniques are inhibited by the small size and fragility of neonatal blood vessels, especially in preterm patients [2]. These factors make UCs the best tool for facilitating fast, life-saving access soon after birth, as well as for stable, low-risk, and pain-minimizing central access in the following weeks of life [3].

Umbilical catheters are positioned within the vasculature based on anatomic landmarks: for high-lying UACs, the catheter tip is positioned between thoracic vertebral bodies T6-9; for low-lying UACs the tip is between lumbar vertebral bodies L3-5; and for UVCs, the tip is positioned at the inferior cavoatrial junction (radiographically landmarked as at the diaphragm). Before placement, the approximate depth of UC placement is calculated using a variety of methods [4, 5]. UC tip position is confirmed by a combined anterior-posterior chest and abdominal x-ray at the time of placement, electrocardiography, or, increasingly, by ultrasound, and by intermittent follow-up studies to check that the catheter tip remains in proper position [6–8]. While external catheter position at the insertion site is periodically visually monitored by the clinical care team, this approach may be unreliable due to the desiccation of the Wharton’s jelly post-insertion and inconsistencies between staff; as a result, radiographic assessment remains the gold standard in assessing UC position.

While UCs have benefits, they are not without risk. Risks of UCs are numerous: catheter migration culminating in line removal or replacement, central line-associated bloodstream infection (CLABSI), thrombosis, pleural or pericardial effusion, cardiac tamponade, and infiltration. Systematic reviews and meta-analyses demonstrate UVC and UAC migration rates of 36.7% and 0.5%, CLABSI rates of 3.9% and 0.4%, and thrombosis rates of 6.5–12% and 8.2%, respectively, while cardiac complications from UVCs have broadly been identified as having rates of 0.3% [9, 10]. The remaining previously described complications are considered rare and documented through numerous case studies but lack comprehensive reports of incidence rates. Since some of these complications are linked to UC migration and malposition, NICUs may elect to discontinue UCs, either prophylactically or electively, often requiring a subsequent central catheter. In the instance of UVCs, this is usually a peripherally inserted central catheter (PICC), while in the case of UACs, it is often a peripheral arterial line (PAL). Each of these means of access carries associated risks.

To prevent bloodstream infections, guidelines from the Centers for Disease Control and Prevention (CDC) advise that UCs be removed as soon as medically possible, with 5 days maximum for UACs and 14 days maximum for UVCs [11]. A more recent guideline has suggested the removal of UVCs after 7 days [12]. These recommendations are based on three publications, with data sets collected before 1994, in 1997, and in 2014, when using adhesive-based securement was standard of care and before the development of CLABSI reduction bundles that gained widespread acceptance in the second decade of the 2000s [13–15]. Levit et al. showed an average UVC dwell time of 9.8 days at the time of a CLABSI, while additional studies show a 3–5 times increase in risk after 7 days [16–18]. Meanwhile, a survey of 72 NICUs in the UK showed 51.5% of units remove UVCs on day 7 or earlier, and a survey of 19 NICUs in Australia and New Zealand showed 52.6% of units remove UVCs before day 7 [19, 20]. In practice, dwell times are chosen based on an assessment of risks and benefits and may be briefer than these maximums due to malposition using current securement methods or concerns for future malposition and complications.

**Fig. 1:**
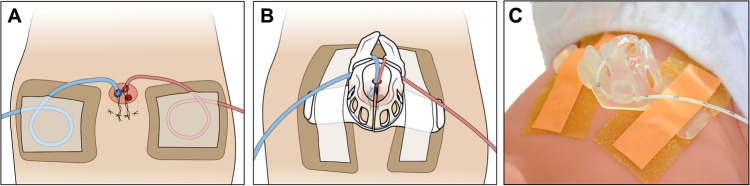
Umbilical catheter securement methods. **A** Adhesive control (AC) method of securing umbilical catheters. **B** LifeBubble (LB) method of securing umbilical catheters. **C** Photograph of the LifeBubble (LB) device for securing umbilical catheters.

It follows that UCs are among the most challenging catheters in medicine to secure due to their insertion through desiccating tissue, their angle of insertion into the vessel, the potential for one or two catheters to be placed and removed at different times, and the relatively small area with which catheters can be secured. Common UC securement techniques include suturing the catheter into Wharton’s jelly and the umbilical stump or skin (depending on local practice), with the addition of a transparent dressing that adheres to the catheter and the patient abdomen and can occlude the umbilical insertion site, “tape bridges” or “goal posts”, and adhesives (Fig. 1A) [21]. While versatile, these methods are those used in publications demonstrating frequent catheter tip migration. These methods also leave the insertion site uncovered to facilitate desiccation, potentially allowing bacterial exposure during infant care and from diapers. Compared to the sterile, standardized solutions required for central catheter use at alternative insertion sites in neonates (PICCs, PALs) and in children and adults (PICCs, PALs, and CVLs), clinicians remain limited in their options to protect patients from UC complications.

We investigated outcomes associated with the use of a new device designed specifically for UC securement, LifeBubble (Laborie, Portsmouth, NH, USA). The device secures the catheters while also protecting the insertion site. The study aims to determine if the device impacts rates of UC tip migration, malposition leading to discontinuation, and CLABSI, when compared to historical control. The device is a small sterile silicone dome incorporating a cleat and strap that is adhered to the patient’s abdomen (Fig. 1B, C), protecting the insertion site while allowing for aeration, desiccation, and visualization of the catheters and insertion depth markers [22].

## Methods

A retrospective concurrent chart review was performed on infants requiring UCs at Oregon Health & Science University’s Level IV NICU from 2019–2021. The study was approved by the OHSU Institutional Review Board (IRB) # 20364. The study population was composed of patients requiring at least one UC during this period; all umbilical catheters for infants in this study were placed on the first day of life. The choice of a LifeBubble or standard securement was at the discretion of the practitioners placing the catheters (Fig. 1A–C). Standard securement (adhesive group) consisted of a suture anchored into the umbilical cord stump, then snugly wrapped around the catheter and knotted, then adhered to the abdomen with a transparent dressing (Tegaderm, 3 M, Maplewood, MN, USA) atop a hydrocolloid dressing (DuoDERM, ConvaTec, Reading, UK) (Fig. 1A). LifeBubble securement (LifeBubble group) consisted of the LifeBubble device applied per manufacturer recommendations by mechanically securing the catheter and adhering the product to the patient with zinc oxide adhesive atop a hydrocolloid skin barrier (Fig. 1B, C); the same suturing technique was also used in the LifeBubble group.

Inclusion criteria included infants ≥1 kg with at least one UC and an x-ray demonstrating final catheter tip position with accompanying documentation in the medical record of the final position of the UC(s). The LifeBubble available at the time was not used in infants <1 kg due to the size of the device; since this study, a smaller device designed for infants <1 kg has been made available. Exclusion criteria included infants who weighed <1 kg, those who were deceased or transferred out of the OHSU NICU before UC removal, and infants without an x-ray documenting final UC position. UCs were removed for one of three reasons: 1 - they were no longer needed; 2 - due to malposition following migration; or 3 - when, at the discretion of the care team, the catheter(s) required replacement due to age per CDC recommendations for CLABSI reduction in the NICU [11, 12].

Patient demographics were collected as shown in Table 1. Outcome measures were 1 - the proportion of UCs that migrated more than one vertebral body in or out as assessed by x-ray establishing the initial catheter position, followed by subsequent x-rays until the UC was removed; 2 - The proportion of UCs that were removed due to malposition following migration; and 3 - CLABSIs. The position of UCs on X-rays was determined by two independent reader types, one being the attending pediatric radiologist reading the film and the other being a single neonatologist on the study team (BS). For UVCs, malposition was defined as the catheter tip being below the diaphragm, resulting in removal by the clinical team. For UACs, malposition was defined as the catheter tip being below T9, resulting in removal per the clinical team. CLABSI was defined as a positive bacterial blood culture (BD Bactec, Becton Dickenson, Franklin Lakes, NJ, USA) within 48 h of blood inoculation that was associated with an indwelling umbilical catheter of either type.

**Table 1:** Patient demographics in each group.

Characteristic	Adhesive control (AC) (*n* = 61)	LifeBubble (LB) (*n* = 57)	*P*-value
Female, *n* (%)	32 (52.5)	23 (40.4)	0.188^b^
Birth weight, g, mean (std dev)	2150 (980)	2598 (1026)	0.016^a^
Gestational age at birth, weeks, mean (std dev)	33.4 (4.6)	34.9 (4.1)	0.064^a^
Congenital heart disease, *n* (%)	18 (29.5)	27 (47.4)	0.046^b^
Total umbilical catheters (UCs), *n*	104	85	–
Patients with two umbilical catheters, *n* (%)	43 (70.5)	28 (49.1)	0.018^b^
Final placement film, *n* (%)	57 (93.4)	50 (87.7)	0.351^b^
Central line associated blood stream inflection (CLABSI), *n* (%)	0 (0)	0 (0)	–
Female, *n* (%)	32 (52.5)	23 (40.4)	0.188^b^

^a^By Wilcoxon Rank Sum test.

^b^By Pearson’s X^2^ or Fisher’s Exact test.

Stata v14 (College Station, TX, USA) was used for statistical analyses. Wilcoxon Rank Sum, Student’s t-test, Pearson Chi Square, and Fisher’s Exact tests were used to evaluate differences in demographics and UC measurements, as appropriate. Logistic regression was used to evaluate incidence of UC (combined UAC and UVC) movement by one vertebral body or more, UVC movement by more than one vertebral body, and UVC discontinuation because of malposition due to migration, UAC movement by more than one vertebral body, and UAC discontinuation because of malposition due to migration between the two groups. Outcomes found to be statistically significant on unadjusted univariate analyses (birth weight, gestational age at birth, presence of congenital heart disease, and presence of two concurrent UCs) were compared using multiple logistic regression to adjust for the potential confounding effects of these significant variables.

## Results

There were 171 patients who had UCs placed during the retrospective review period. 43 patients were <1 kg and were excluded from the review; 10 patients did not have a final film establishing UC position following adjustment of catheter position. Study criteria resulted in 61 and 57 patients in the adhesive and LifeBubble groups, respectively (Table 1). Between groups, there were significant differences in rates of congenital heart disease diagnoses (47.4% vs 29.5%, *p* = 0.046), presence of two concurrent UC catheters (49.1% vs 70.5%, *p* = 0.018), and in birth weight (2598 g vs 2150 g, *p* = 0.016) in the LifeBubble group versus the standard adhesive control group. There was also a non-significant trend towards a difference in gestational age at birth (Table 1). No other significant variation was found in patient demographics between study groups.

Of the 188 UCs studied, 104 were secured with adhesive (54 UVC, 50 UAC) and 85 were secured with LifeBubble (50 UVC, 35 UAC) (Table 1). There were no disagreements between the pediatric radiology and neonatology reading of UC positions. Comparing UC securement of the LifeBubble group to the adhesive group (Table 2), and after adjusting for potential confounding effects of birth weight, gestational age, two versus one umbilical catheter, and congenital heart disease, we found significantly fewer instances of UC migration of more than one vertebral body (12.3% vs 55.7%: *p* < 0.001). When focusing on UVC position, we observed significant reductions in UVC migration of more than one vertebral body (5.3% vs 39.3%; *p* = 0.001) and UVC migration leading to discontinuation (5.6% vs 37.7%; *p* = 0.002). Based on these absolute risk reductions, the number needed to treat (NNT) to prevent 1 instance of UVC migration of more than one vertebral body is 3 and the NNT to prevent UVC discontinuation due to malposition is 4. There were also significantly fewer instances of UAC migration (7.0% vs 23.0%, *p* = 0.01) in the LifeBubble group compared with the adhesive group; UAC discontinuation was less frequent in the LifeBubble group (0% vs 6.6%, *p* = 0.119), but these findings were not statistically significant. No CLABSIs occurred in either group.

**Table 2:** Catheter migration and early discontinuation in each group.

Outcome measure	Adhesive control (AC) (*n* = 61))	LifeBubble (LB) (*n* = 57)	*P*-value	Adjusted *P*-value
Umbilical catheter moved more than one vertebral body, *n* (%)	34 (55.7)	7 (12.3)	<0.001^a^	<0.001
UVC movement more than one vertebral body, *n* (%)	24 (39.3)	3 (5.3)	<0.001^a^	0.001
UVC discontinuation, *n* (%)	23 (37.7)	3 (5.6)	<0.001^a^	0.002
UAC movement more than one vertebral body, *n* (%)	14 (23.0)	4 (7.0)	0.022^a^	0.01
UAC discontinuation, *n* (%)	4 (6.6)	0 (0)	–	–

^a^By Fisher’s Exact test, as appropriate.

## Discussion

This study aimed to quantify the impact of a novel device-based approach to UC securement and stabilization on rates of UC migration, malposition leading to premature discontinuation, and CLABSI. Here, we found a significantly lower incidence of UVC migration leading to discontinuation, as well as significantly fewer instances of UC migration overall, in the LifeBubble group compared to the adhesive control group. These findings demonstrate that the LifeBubble device can provide a significant clinical advantage in the prevention of UC migration and migration leading to early discontinuation. This report is the first to demonstrate the potential of an intervention to increase UC stability in neonates.

UCs are an essential tool for medication delivery and monitoring in neonates, yet high complication rates exist in part due to the lack of reliable and standardized securement methods, and because of the unique aspects of UCs when compared to other catheters: placement into vessels end on and the fact that these vessels are part of a non-vital fetal remnant that becomes desiccated. The only other comparable conditions for central access in medicine are in serious burn patients, which can require catheter placement through devitalized tissue. Studies for such central catheters demonstrate CLABSI rates of 15.4–20.6 per 1000 catheter days [23, 24].

The decrease in UVC migration incidence seen in this study from 39.3% (adhesive control group) to the 5.3% (LifeBubble group) (Table 2) remained statistically significant even when adjusting for significant differences in the two groups, including birth weight, gestational age at birth, presence of both UCs, and congenital heart disease. The same is true for the decrease in UAC migration (23.0% adhesive control to 7.0% LifeBubble) when adjusted for differences in the two groups, As might be expected from the individual UVC and UAC migration results, the reduction in combined UC migration incidence was also statistically significant, with the reduction likely driven more by the effect on UVC migration. Notably, the number needed to treat to prevent UVC discontinuation in this study is only 4, an impressively low number that suggests that use of the LifeBubble device could have a significant impact on clinical care.

Migration may be a cause of CLABSIs in NICU patients. UC sites are currently unprotected and non-sterile. If a catheter exposed to this bacteria-prone environment migrates deeper, bacteria on the catheter surface could be directly carried into the patient’s bloodstream, however, UC migration inward and linkage to increased risk for CLABSI has yet to be demonstrated. While this study did not include any CLABSIs to report, such hospital-associated infections remain a key focus of quality improvement metrics across the US healthcare system. Catheter migration and dislodgement under the current standard of care may lead to variations in NICU policies for family holding and prone positioning with UCs, as well as variations in practice between providers or nursing staff within a NICU.

The ability to prevent migration and discontinuation could also impact dwell time and the need for short-term alternative central catheters. These replacement catheters come with their own risks at initial insertion and throughout use. Commonly used PICCs have been shown to have increasing CLABSI risks after the first 14 days of use [25]. Decreased UC migration has the potential to lead to increased maximum dwell times, which could extend through the end of a patient’s highest acuity period and eliminate the need for additional central catheter insertion. In some patients, a second central catheter may be entirely avoided with increased UVC dwell time.

The decrease in umbilical catheter migration, particularly UVC migration, would likely directly translate, over many patients, to the prevention of serious adverse events in these neonatal patients. Intracardiac migrations can be fatal due to complications including intracardiac thrombosis and myocardial perforation, while migrations outward can lead to severe liver damage [26]. A larger study with more patients, powered to detect a difference in these rarer life-threatening effects, would be necessary to assess the potential prevention of such downstream complications.

The results of this study, as well as past studies, demonstrate lower migration risks associated with UACs when compared with UVCs [10]. While the reason for this is not firmly established, generally UACs are used for monitoring blood pressure and arterial blood gasses, while UVCs are used for the active delivery of fluids, nutrition, and medications. This may result in increased frequency and duration of UVC handling, leading to increased opportunity for catheter movement. Additionally, the larger region of appropriate positioning for UACs (T6-9) means that movement is less likely to lead to mispositioning. In the future, these UC dwell times could both be safely extended based on the ability of new technology to enable catheter securement and insertion site protection while limiting complication risks.

Patients in the LifeBubble group also had lower overall UC migration rates despite the significant difference in congenital heart disease patients. Congenital heart disease patients have been shown to be at higher risk for UVC complications [1, 27]. This could be due to more extensive testing and procedures that make a patient more prone to movement, longer durations of time in which central catheters are needed, the anatomy of the heart itself, or the physical movement of the heart itself in patients undergoing cardiac repair.

A leading reason for the success of the LifeBubble group is likely the mechanism of mechanical securement. Adhesives are prone to failure after exposure to moisture or repeated/excessive movement. Meanwhile, the LifeBubble uses a silicone strap to lock the UC into place without evidence of impeded catheter flow. The insertion site is also protected by the body of the device, while vents allow for stump desiccation and visualization [22, 28]. Furthermore, standardization has been recognized as a key component to quality improvement and patient safety [29–31]. The current standard of care for UC securement and maintenance requires subjective behavior of the clinical team which is prone to variation in system stability and resulting clinical outcomes.

There was a statistically significant difference in birth weights between the standard adhesive securement and LifeBubble groups, with larger birth weights in patients in the LifeBubble group. This was due to the non-randomized nature of the study and apparent bias in the choice of securement method. While this difference does not impact the conclusions drawn from the study results by logistic regression, a smaller shift in catheter positioning will have a proportionally larger impact on patients of lower birth weight. Lower birth weight patients may therefore be more likely to experience clinically significant migration from small catheter movement, emphasizing the importance of umbilical catheter position stability. The relatively low proportion of patients classified as very low birth weight (<1500 g) and the exclusion of extremely low birth weight (<1000 g) patients are relevant limitations of the study. The efficacy of the LifeBubble device in such populations requires additional research.

There are limitations to this study and the reported results. First, the study design was single center and retrospective, and the choice of securement method was not randomized, which introduces the possibility of bias into the results. The study reports pragmatic results based on current routine care, in which the control group uses a standard practice of adhering the UC to the abdomen. Both this adhesive method and the alternative tape in a goal post formation are considered standard practice for NICUs around the globe. An ideal study design would be both prospective and with randomization of the securement method. There was a significant difference in mean birth weight with a slightly higher birth weight in the LifeBubble group. The larger birth weight in the LifeBubble group may have had a tendency to reduce the rate of UC movement and malposition leading to discontinuation, but not enough to affect the significance of the reductions in the outcome measures of UVC movement and malposition leading to discontinuation and UAC discontinuation with LifeBubble securement compared to standard adhesive securement. Furthermore, this study does not address the meaningful group of premature neonates with birth weights <1000 g. While these patients are at risk for UC migration and infection complications, the LifeBubble product available at the time of the study was not used for extremely low birth weight patients due to its size.

To date, umbilical catheter migration has been largely viewed as an unavoidable and intrinsic disadvantage of these central catheters. Securement of umbilical catheters is a chronic problem and the current securement techniques need to be improved upon to reduce rates of UC malposition. A recent study evaluated the potential of using cyanoacrylate glue to reduce UVC dislodgement rates in the first 48 hours [32]. Solutions are evolving, and this study demonstrated one way for NICUs to decrease early UC discontinuation rates and improve resulting critical patient outcomes, through the adoption of a standardized, mechanical securement technique such as the LifeBubble device.

## Data Availability

Inquiries regarding the raw data included for analysis can be directed to BS.
